# Access to DTP-Based Combination Vaccines in Asia–Pacific Countries between 2019 and 2022

**DOI:** 10.3390/vaccines12010033

**Published:** 2023-12-28

**Authors:** Jiyan Ma, Yinuo Sun, Yuxuan Cui, Jingya Dong, Yangmu Huang

**Affiliations:** Department of Global Health, School of Public Health, Peking University, Beijing 100191, China1810306234@pku.edu.cn (Y.C.);

**Keywords:** health access, diphtheria, tetanus, pertussis, vaccine, Asia-Pacific, COVID-19

## Abstract

The Asia–Pacific countries are highly diverse in health and economic conditions that may impact vaccine access and uptake. Our study aimed to characterize patterns of health access to DTP-based combination vaccines in 10 countries from 2019 to 2022 using the IQVIA-MIDAS database. The availability, affordability, and accessibility were compared across countries by national health and economic performance indicators using Spearman’s rank correlation coefficient. Our findings showed that the three aspects of access to DTP-based vaccines varied substantially in the Asia–Pacific region, with higher levels in countries with better health and economic performance. Affected by the COVID-19 pandemic, vaccine accessibility fluctuates significantly in lower-income countries, with DTP coverage rates falling by more than 14% in the Philippines and Indonesia between 2019 and 2021. For availability and affordability, Singapore and Malaysia from high-income groups were largely affected, which may be related to health expenditure as a percentage of gross domestic product (Coefficient = 0.39, *p* = 0.03). Our study indicates that greater attention needs to be paid to national health expenditure and routine immunization services to improve vaccine disparities and increase the robustness and resilience of the vaccine supply chain during public health emergencies.

## 1. Introduction

With the improvement of essential health services, the types and doses of vaccines available to children at an early age are being increasingly introduced. Combination vaccines provide an ideal solution to reducing children’s pain and discomfort from numerous injections by administering multiple strains or serotypes of antigens simultaneously. As the most commonly used combination vaccine, the diphtheria-tetanus-pertussis (DTP) vaccine plays an essential role in reducing overall childhood mortality from three fatal diseases. Driven by vaccine technology, products are available as a single DTP vaccine and in combination with other antigens, such as polio, hepatitis B, and Haemophilus influenzae type b (HiB). The DTP-based vaccine has been included in part of the Expanded Programme of Immunization (EPI) since 1974 and is regarded as a measurement subject for routine immunization performance across different countries [1].

Despite the recognized benefits, there is a persistent challenge of global disparities in DTP vaccine use and coverage, associated principally with access to health services [2]. In the Asia–Pacific (APAC) region, the National Immunization Programs (NPIs) vary across countries in terms of vaccine types, timing, frequency based on local morbidity and mortality, and cost-effectiveness analysis. For example, high-income countries, such as Australia and New Zealand, use DTP combination vaccines based on acellular pertussis (aP), which is more expensive and has fewer adverse events, whereas India and Vietnam with lower income levels are consistent with World Health Organization (WHO) recommendations on whole-cell pertussis (wP) [3]. These inequalities were exacerbated disproportionately during public health emergencies due to inadequate investment, insufficient health infrastructure, and unaffordable prices [4]. Between 2019 and 2021, the proportion of children completing the primary immunization series of DTP vaccines decreased by 5% globally, the most significant decline in three decades [5]. The APAC region is the most affected area, experiencing a 9% decline in vaccine coverage [5]. Due to the heavy burden of communicable diseases among children in most APAC countries, the coverage decline could put millions of children at risk of vaccine-preventable diseases [6]. Understanding the severity of the disruption and potential factors is urgently needed for securing a resilient global vaccine supply chain.

Few studies have examined the trend and pattern of access to DTP-based vaccines between countries with different levels of health and economic development, particularly in the APAC region. There are scarce findings regarding the extent and determinants of the disrupted supply of routine immunization services affected by the COVID-19 pandemic. Our study conducted a multinational cross-sectional study of health access to DTP-based vaccines across ten APAC countries between 2019 and 2022. We aimed to explore the inequality and disruption of three access indicators (including availability, affordability, and accessibility) for DTP-based vaccines during the pandemic and examine whether the access affected by COVID-19 was associated with country health and economic performance. Our findings hoped to identify potential factors and approaches to strengthen the supply chain resilience of essential childhood vaccines during public health emergencies and inform global actions on equitable access to routine vaccination services for preparedness and response to future outbreaks. 

## 2. Materials and Methods

### 2.1. Study Setting

We selected ten countries for our study based on the following criteria: (1) These countries have included the DTP vaccine in the NIPs; (2) available country sale data could be accessed in the IQVIA-Multinational Integrated Data Analysis System (MIDAS) database; and (3) these countries are diverse in disease burden, health system performance, and income level to make comparisons more meaningful. Indonesia, Malaysia, New Zealand, Pakistan, the Philippines, Singapore, Thailand, Vietnam, India, and Australia were included in our study. The characteristics of these ten countries are presented in Table 1.

### 2.2. Data Source and Collection

Our data were mainly derived from quarterly estimates of DTP-based vaccines sold in these countries between 2019 Q3 and 2022 Q2 from the IQVIA-MIDAS database. The database provides standardized estimates on the sale volume and value of pharmaceutical products in 93 countries using national sample surveys throughout the whole distribution channel (i.e., from manufacturers to wholesalers to retailers, hospitals, pharmacies, and clinics) [7]. It has been validated against external data sources and used for multinational analyses of health access and utilization of various pharmaceutical products [8,9,10]. The database contained information on product name, country, setting, manufacturer name, time, strength, formulation, sale volumes in standard units (SU), and sale values in United States dollars (USD). A SU is defined as the smallest common dose of a product form, such as a single tablet or one dose of pre-filled injection, which enables direct comparison of DTP-based vaccines sold in different countries. We searched for DTP-based vaccines using a combination of subject terms: “vaccine”, “diphtheria”, “tetanus”, and “pertussis”. All DTP-based combination vaccines against these three diseases and above were included in our analysis. 

#### 2.2.1. Vaccine Consumption (Availability)

We assumed sale volumes of DTP-based vaccines obtained from the MIDAS database as a proxy for total national consumption each quarter, following the previous studies [8,10,11,12]. The average consumption per 1000 infants under one year old was calculated to compare the number of available vaccines that can be delivered to infants in need (or vaccine availability) across different countries. The population data for each country from 2019 to 2022 were obtained from the World Bank database.

#### 2.2.2. Vaccine Expenditure (Affordability)

We assumed the sale values of DTP-based vaccines sold in these countries to be equal to the total national expenditure each quarter. The average expenditure per 1000 infants was measured to estimate financial resources spent on DTP vaccines for infants under 1 year old in each country (or vaccine affordability). The dollar values were reported in the year they occurred. 

#### 2.2.3. Vaccine Coverage Rate (Accessibility)

To assess the extent of reach of DTP-based vaccination services in infants at the national level (or vaccine accessibility), we extracted the DTP 1 and DTP 3 coverage rates of ten countries between 2010 and 2022 from the WHO/UNICEF Estimates of National Immunization Coverage (WUENIC). DTP 1 and DTP 3 are defined as the percentages of one-year-old infants who received the first and third doses of the DTP vaccine in a given year [12]. They are crucial indicators for measuring NIP performance in initial and sustainable engagement for vaccinating the target population. 

#### 2.2.4. National Health and Economic Performance 

The APAC countries were classified by income level based on the World Bank Country Group Classification as low-middle-income countries (LMIC, Gross National Income [GNI] per capita between USD 1136 and USD 4465), upper-middle-income countries (UMIC, GNI per capita between USD 4466 and USD 13,845), and high-income countries (HIC, GNI per capita greater than USD 13,846) [13]. We selected the following measures for each country: (1) Gross Domestic Product (GDP) per capita; (2) incidence and mortality rates of diphtheria, tetanus, and pertussis among children under one year old; (3) current health expenditure per capita; (4) current health expenditure as a percentage of GDP; and (5) Universal Health Coverage (UHC) service coverage index. Data were derived from the International Monetary Fund, the Global Burden of Diseases, Injuries, and Risk Factors Study, the World Bank, and WHO databases. All expenditures were expressed in current USD.

#### 2.2.5. Statistical Analysis

Descriptive statistics were summarized using the mean and interquartile range (IQR). The data for 2019 Q3–Q4 and 2022 Q1–Q2 were extrapolated to obtain the subject year’s level by doubling the sum of two quarters. To explore the disruption and recovery of DTP-based vaccine access affected by the COVID-19 pandemic, we calculated the difference between three access indicators from 2019 to 2021 and from 2021 to 2022 as the global routine immunization service gradually recovered in 2022 [5]. Correlations between access and health and economic performance indicators were examined using Spearman’s rank correlation coefficient. To explore the influencing factors of each access indicator in terms of overall and fluctuation levels affected by the pandemic, we analyzed the annual absolute number and relative change of vaccine consumption per capita, vaccine expenditure per capita, and coverage rate per year at the individual country level between 2019 and 2022. The statistical significance level was set at a *p* value less than 0.05. We used SPSS Statistics 29.0.10 (Chicago, IL, USA) for all statistical analysis.

## 3. Results

### 3.1. Availability of DTP-Based Vaccines

The average consumption of DTP-based vaccines varied exponentially among these countries, ranging from 76.8 SU in the Philippines to 812,339.8 SU in India between 2019 and 2022. Countries with the highest levels of average consumption per 1000 infants were HIC and UMIC, namely Singapore (7664.3 SU/per 1000) and Malaysia (4079.4 SU/per 1000) (Table 2). In contrast, the Philippines and Pakistan from the LMIC group accounted for the lowest levels of 0.1 and 9.9 SU/per 1000.

**Figure 1:**
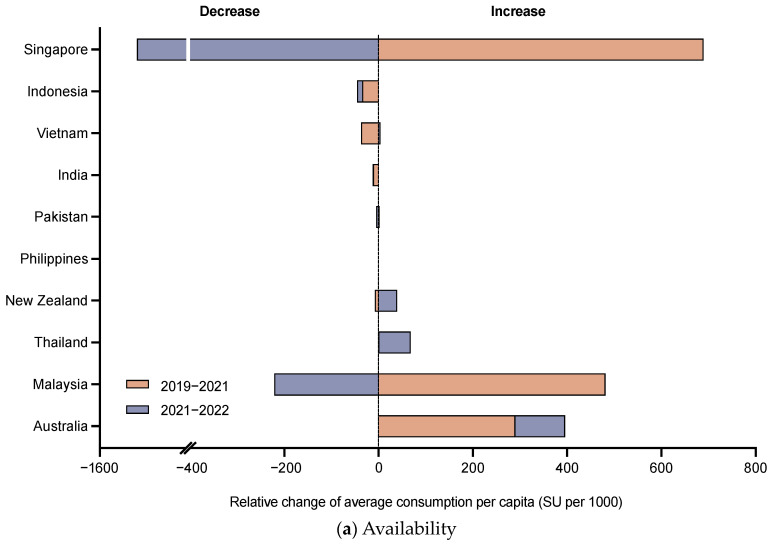

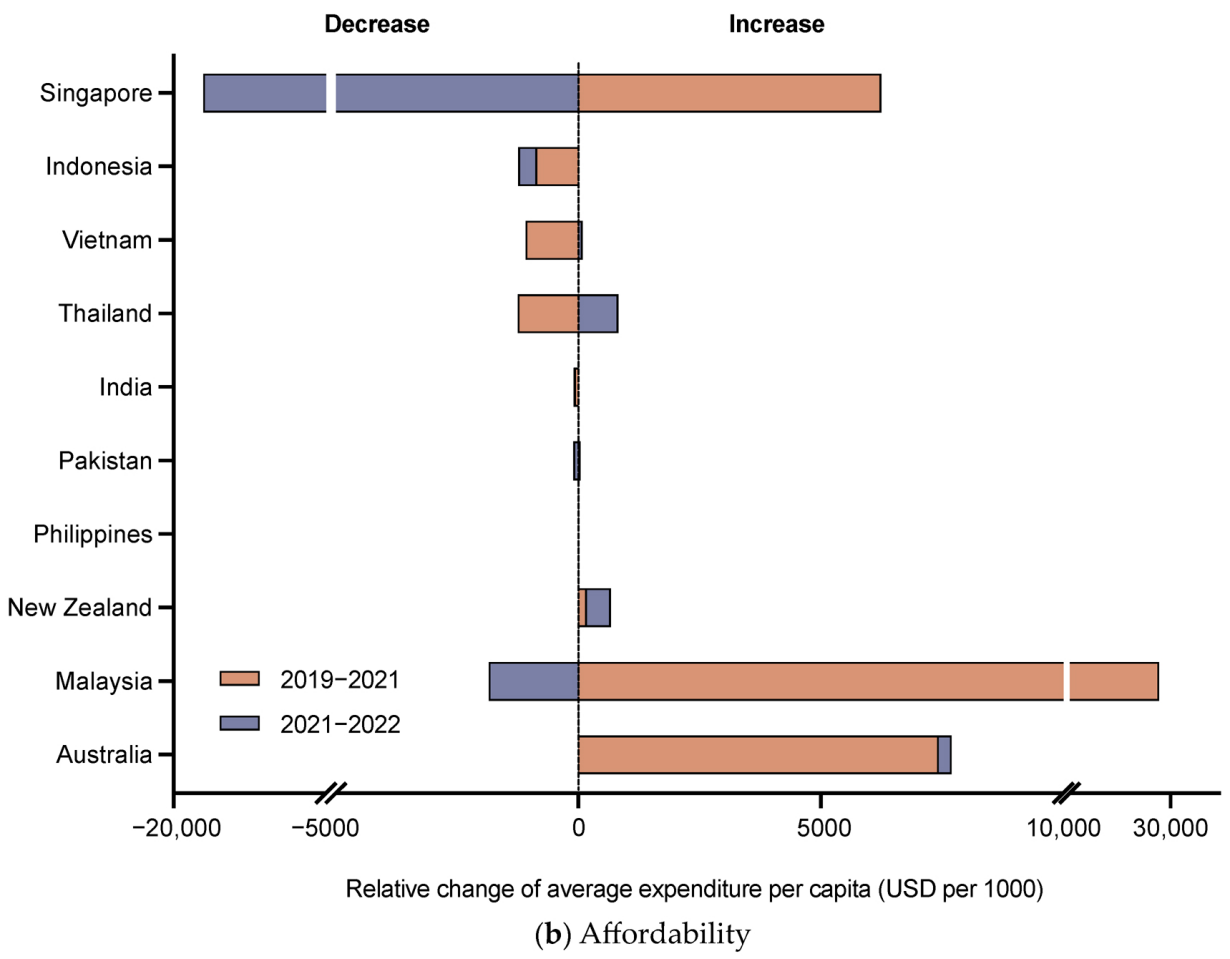
(**a**) Relative changes of average consumption per capita between 2019 and 2022 by country; (**b**) Relative changes of average expenditure per capita between 2019 and 2022 by country. Relative change depends on the difference between the start and end years. For example, the 2019 data were used as the baseline for determining the relative change from 2019 to 2021.

Between 2019–2021 and 2021–2022, the availability of DTP-based services affected by the COVID-19 pandemic varies across countries (Figure 1a). Australia and Thailand demonstrated sustained increases of 396.2 and 68.1 SU/per 1000. Philippines, India, and Indonesia declined consecutively, but with relatively small degrees of 0.03, 13.1, and 45.2 SU/per 1000. Malaysia first increased between 2019 and 2021, but decreased afterward. In addition, the vaccine availability in three countries (i.e., Vietnam, Pakistan, and Singapore) has not yet recovered to the pre-pandemic level, with the most decline in Singapore (395.3 SU/per 1000 between 2019 and 2022).

### 3.2. Affordability of DTP-Based Vaccines

The patterns of average expenditure and average expenditure per 1000 infants of DTP-based vaccine across countries were similar to those of consumption. Philippines (2466.1 USD) and India (14,701,081.6 USD) hold the lowest and highest sale values, respectively. The average expenditure per 1000 infants in countries with higher income levels, such as Singapore (138,398.5 USD/per 1000) and Malaysia (58,632.7 USD/per 1000), outweighs those in LMIC groups, for instance, the Philippines (4.2 USD/per 1000) and Pakistan (161.1 USD/per 1000) (Table 2). For vaccine affordability during the COVID-19 pandemic, Australia and New Zealand were not affected, maintaining an increase of 7700.1 and 671.3 USD/per 1000 (Figure 1b). Philippines, India, and Indonesia declined consecutively by 1.1, 99.4, and 1234.8 USD/per 1000. There are also four countries whose levels have not returned to the pre-pandemic level, including Pakistan, Thailand, Vietnam, and Singapore. Singapore has fallen the most by 7.5% (10,834.3 USD/per 1000).

### 3.3. Accessibility of DTP-Based Vaccines

**Figure 2:**
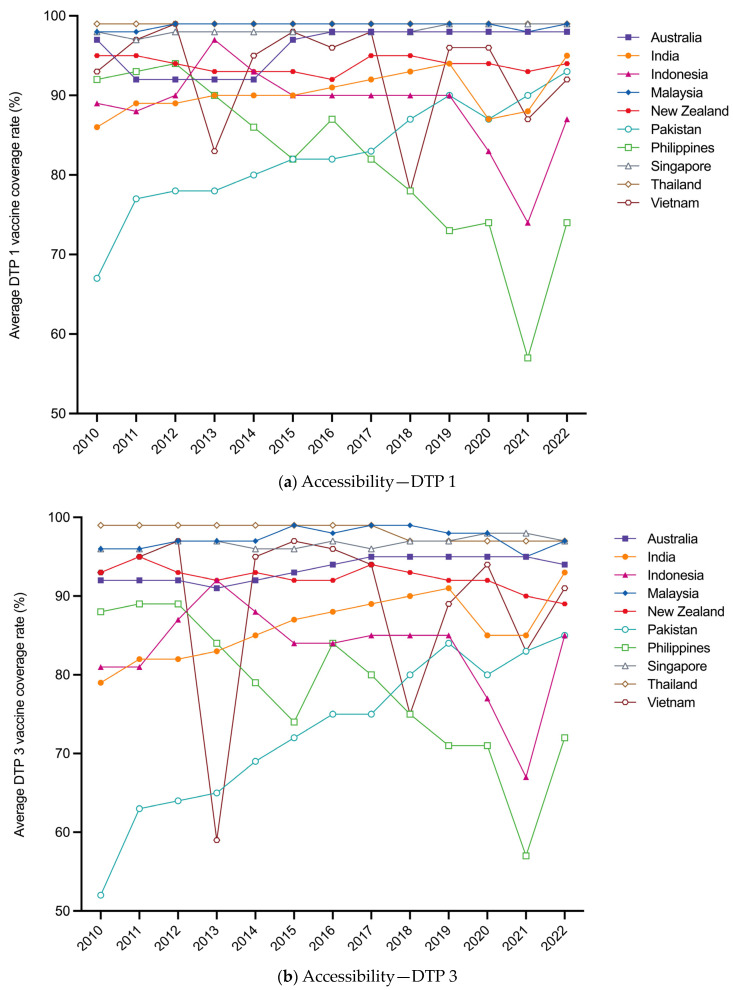
(**a**) Pattern of average DTP 1 coverage rate between 2010 and 2022 by country; (**b**) Pattern of average DTP 3 coverage rate between 2010 and 2022 by country.

From 2010 to 2022, five out of ten countries have reached the global target of 90% for average DTP coverage rate, including Thailand (DTP 1 and DTP 3: 99.0% and 98.2%), Malaysia (98.8% and 97.4%), Singapore (98.2% and 96.8%), Australia (96.0% and 93.5%), and New Zealand (93.8% and 92.3%). Pakistan (82.6% and 72.8%) and the Philippines (81.7% and 77.9%) lag behind other countries on average, but Pakistan continued to increase by more than 20% during the 2010–2020 period and the Philippines significantly decreased by more than 15%. After the outbreak of COVID-19, the DTP coverage rates in most countries experienced varying degrees of decline between 2019 and 2021 and rebound between 2021 and 2022. The lower-income group fluctuated greatly, represented by the Philippines and Indonesia. For DTP 1, Vietnam and Indonesia have not yet returned to pre-pandemic levels, falling by 4% and 3%, respectively (Figure 2a). For DTP 3, New Zealand and Australia remained stable at first but later declined, and Malaysia has not recovered, falling by 1% (Figure 2b). 

### 3.4. Correlation between Access to DTP-Based Vaccines and National Health and Economic Performance

To explore the overall correlation between national performance and health access and the relationship between national performance and the fluctuation of health access during COVID-19, we analyzed the annual absolute number and relative change of three access indicators between 2019 and 2022, as described in Section 2.2.4 and Section 2.2.5. At the overall level, significant correlations were observed between DTP-based vaccine access and most national health and economic performances (Table 3). The incidence and mortality rates are negatively correlated with vaccine access, while the other national performance indicators are positively correlated. The current health expenditure as a percentage of GDP is only significantly correlated with average consumption per capita (Coefficient = 0.417, *p* = 0.007) and unrelated to affordability and accessibility. However, in terms of fluctuation, almost all national development indicators were independent of relative changes in DTP-based vaccine access during the outbreak. Only the current health expenditure as a percentage of GDP has positive relationships with the fluctuation of per capita consumption (Coefficient = 0.393, *p* = 0.032) and expenditure (Coefficient = 0.389, *p* = 0.033), indicating that the greater the proportion of national investment in health, the more obvious the change in DTP vaccine availability and accessibility affected by the pandemic.

## 4. Discussion

Our study provides the first and most up-to-date investigation of DTP-based vaccine access in APAC countries before and after the COVID-19 pandemic. The main findings reveal that vaccine availability, affordability, and accessibility are significantly related to GDP per capita, disease burden, health expenditure per capita, health expenditure as a percentage of GDP, and the UHC service coverage index. Access to DTP-based vaccines was affected by the pandemic to varying degrees across countries, with countries with high proportions of health expenditure in GDP being more affected. Enhancing national health expenditure and routine immunization services could help facilitate DTP-based vaccine access and improve the robustness and resilience of the vaccine supply chain for future outbreaks.

An effective NIP is to ensure the continuous and uninterrupted provision of essential vaccines up to the point of vaccination. Missed opportunities incurred by interrupted supply may increase the incidence of vaccine-preventable diseases among children. More recent evidence points to the fact that the vaccine supply chain has gradually outgrown its ability to maintain continuous and uninterrupted availability of routine childhood vaccines [14,15,16]. DTP-based vaccines, as one of the most affected products, reported 43% of stockout events in a previous study of 17 countries [14]. The inconsistent supply of DTP vaccines in the Philippines reduced the national coverage rate by 15%, leading to the rise of diptheria-reported cases in 2015 [14]. Our study reflects that the vaccine supply system in HIC and UMIC countries is generally more stable and improved because of the higher level of average consumption and expenditure per capita in Singapore and Malaysia and the continued growth in Australia and Thailand between 2019 and 2022. In contrast, the level of LMICs is relatively low, such as in the Philippines and Pakistan, which might be correlated with inadequate infrastructure, a lack of skilled health workforce, and weak capacity for primary health services. The significant correlation between vaccine access and national performance indicators implies that increasing access to DTP-based vaccines requires not only the efforts of the health sector but also synergies in economic development and financial investment across the country, which was in line with the study of Pierre Muhoza et al. [17]. In addition, apart from national-level indicators, individual- and community-level characteristics (e.g., parental knowledge and vaccine hesitancy) contribute to persistent disparities in vaccine access. The perceived risk of the COVID-19 vaccine was reported as a contributing factor to increased parental vaccine hesitancy about routine vaccinations [18,19]. The dedicated centers or practices for vaccination and educational campaigns for parents could help maintain routine childhood immunization services during the COVID-19 pandemic [20]. Collaboration between governments, health providers, and social media is essential to mitigating the impact of immunization disruptions on the most disadvantaged populations.

The COVID-19 pandemic exacerbated the weaknesses and disparities in the vaccine supply chain. The lockdown and mobility restrictions have severely constrained vaccine production, shipments, and delivery in time, as reported by UNICEF [5]. Significant human and financial resources were diverted to respond to the unprecedented outbreak, resulting in dramatic declines in childhood visits and vaccination coverage. Our study shows that relative change in vaccine access varies greatly across economies, partly associated with different procurement and financing approaches to essential vaccines. The sale volume and value of DTP vaccines are most affected in the HIC group because these countries are heavily dependent on non-local manufacturing pharmaceutical products, and their national healthcare budgets, which primarily supported NIP, were distracted by the pandemic [7]. In contrast, countries in the LMIC group tend to maintain the vaccine supply chain through local manufacturers, Gavi, and other development partners [18,21]. The underlying implications explained the correlation between the proportion of health expenditure in GDP and the relative change in vaccine volume and sale per capita during COVID-19. To improve the access and stability of DTP vaccines, countries need to optimize their policies and resource allocation mechanisms, including increasing investment in health care, optimizing the vaccine supply chain, and establishing more flexible primary health care systems.

Our study is subject to the following limitations: First, it relies on a mix of public and commercial databases. Although these estimates come from the best available sources, there might be subtle differences between different datasets for the same indicators, and data quality issues could result in inaccurate estimations of vaccine consumption, expenditure, and coverage across different countries. Second, due to limited access to the IQVIA database, only ten APAC countries with varying disease burdens, income levels, and health system performances were included in our research. The data were limited to the quarter data from 2019 Q3 to 2022 Q2 by the time of writing, so that the extrapolated annual figures of 2019 and 2022 did not reflect the real situation. However, we observed that the quarter data of individual countries fluctuates slightly in each year during non-pandemic periods, and most countries resumed routine immunization services in the first half of 2022, so that the sum of 2022 Q1 and Q2 reflected most of the recovery trend. Considering the short time frame, the sale values have not been discounted for the inflation rate. Finally, we cannot rule out the competing explanations for the correlation observed as common drawbacks of cross-sectional study design. Other factors that may correlate with health access may not be fully considered and adjusted in the current analysis. Future studies are suggested to investigate the underlying mechanisms of how these factors impact each other. 

## 5. Conclusions

Access to DTP-based vaccines remains unequal and insufficient to protect infants from severe illness and death in APAC countries. We observe significant evidence showing correlations between health access, national health, and economic performance. Increasing the national input on health expenditure and universal health coverage could help reduce vaccine inequalities and ensure a robust supply of essential childhood vaccines during public health emergencies. 

## Figures and Tables

**Table 1:** Characteristics of the 10 APAC countries included in this study.

Characteristic	Australia	India	Indonesia	Malaysia	New Zealand	Pakistan	Philippines	Singapore	Thailand	Vietnam
Income level	HIC	LMIC	UMIC	UMIC	HIC	LMIC	LMIC	HIC	UMIC	LMIC
Population under 1 year old in 2022 (per 1000)	298.5	23,113.5	4496.4	511.3	63.6	6374.7	2485.0	42.2	644.3	1462.6
GDP per capita in 2022 (USD per capita)	64,813.9	2391.9	4798.1	12,465.6	47,226.1	1650.3	3623.6	82,807.6	7069.6	4086.5
Current health expenditure in 2020 (% of GDP)	10.6	3.4	3.0	4.1	10.0	3.0	5.1	6.1	4.4	4.7
DTP vaccine is included in NIP *	DTaP	DTwP	DTwP	DTaP	DTaP	DTwP	DTwP	DTaP	DTwP	DTwP
The incidence rate of diphtheria among children under 1 year old in 2019 (per 100,000)	0.0008	0.1341	0.6005	0.0371	0.0004	0.7591	0.5813	0.0011	0.1007	0.1374
The incidence rate of tetanus among children under 1 year old in 2019 (per 100,000)	0.0008	43.3039	34.7453	0.2974	0.0006	72.5749	11.0156	0.0006	0.2184	3.4995
The incidence rate of pertussis among children under 1 year old in 2019 (per 100,000)	2871	6107	8425	4788	3207	8091	8072	3667	5183	7691

* DTwP is developed with whole-cell pertussis (wP), and DTaP contains the acellular pertussis antigen (aP). Abbreviations: HIC, high-income country; UMIC, upper-middle-income country; LMIC, lower-middle-income country; GDP, gross domestic product; USD, United States dollar; NIP, National Immunization Program. Country income level was classified based on the World Bank Country Group Classification; Population under 1 year old was retrieved from the World Population Prospects of the United Nations; GDP per capita and current health expenditure were from the International Monetary Fund and World Bank databases; the DTP vaccine included in NIP were searched in the WHO Immunization Data Portal; and the incidence rates of three diseases came from the Global Burden of Diseases, Injuries, and Risk Factors Study (2019).

**Table 2:** Availability, affordability, and accessibility of DTP-based vaccines vary by country.

Country	Average Consumption per Capita (SU per 1000) *		Average Expenditure per Capita (USD per 1000)		Average DTP 1 Coverage Rate (%)		Average DTP 3 Coverage Rate (%)	
	Mean	IQR	Mean	IQR	Mean	IQR	Mean	IQR
Australia	922.8	751.1–1097.9	19,105.1	15,321.8–22,485.5	96	92.0–98.0	93.5	92.0–95.0
India	138.7	133.4–144.8	2509.7	2473.4–2555.2	90.3	88.5–92.5	86.1	82.5–89.5
Indonesia	58.6	40.8–78.5	1850.6	1344.6–2379.9	88.5	87.5–90.0	83.2	81.0–86.0
Malaysia	4079.4	3633.7–4450.3	58,632.7	42,371.3–73,938.2	98.8	98.5–99.0	97.4	96.5–98.5
New Zealand	172.8	154.6–195.8	1877.3	1567.7–2261.4	93.8	93.0–95.0	92.3	92.0–93.0
Pakistan	9.9	7.7–11.7	161.1	118.1–200.0	82.6	78.0–88.5	72.8	64.5–81.5
Philippines	0.1	0.1–0.2	4.2	3.1–5.1	81.7	74.0–91.0	77.9	71.5–86.0
Singapore	7664.3	7139.5–8284.1	138,398.5	127,622.4–148,839.5	98.2	98.0–99.0	96.8	96.0–97.0
Thailand	659.4	642.0–693.1	17,711.4	17,194.8–18,306.1	99	99	98.2	97.0–99.0
Vietnam	132.2	98.0–180.6	4126.3	3095.3–5625.5	92.9	89.5–97.5	89.1	86.0–95.5

* Consumption and expenditure are averages from 2019 Q3 to 2022 Q2; the DTP coverage rate is averaged between 2010 and 2022. Abbreviations: SU, standard unit; USD, United States dollar; IQR, interquartile range.

**Table 3:** Correlations between access and national health and economic performance.

Indicators	Average Consumption per Capita		Average Expenditure per Capita		Average DTP 1 Coverage Rate		Average DTP 3 Coverage Rate	
	Overall	Relative Change	Overall	Relative Change	Overall	Relative Change	Overall	Relative Change
GDP per capita								
Coefficient	0.802 **	0.103	0.676 **	0.009	0.641 **	−0.093	0.673 **	−0.076
*p* value	0.000	0.588	0.000	0.964	0.000	0.624	0.000	0.689
Incidence burden								
Coefficient	−0.799 **	−0.302	−0.615 **	−0.25	−0.674 **	0.056	−0.700 **	0.069
*p* value	0.000	0.104	0.000	0.183	0.000	0.771	0.000	0.717
Death burden								
Coefficient	−0.793 **	−0.189	−0.647 **	−0.117	−0.655 **	0.129	−0.684 **	0.097
*p* value	0.000	0.318	0.000	0.537	0.000	0.496	0.000	0.609
Current health expenditure per capita								
Coefficient	0.734 **	0.297	0.572 **	0.231	0.579 **	−0.091	0.608 **	−0.084
*p* value	0.000	0.112	0.000	0.219	0.000	0.633	0.000	0.659
Current health expenditure (% GDP)								
Coefficient	0.417 **	0.393 *	0.269	0.389 *	0.28	−0.01	0.301	−0.028
*p* value	0.007	0.032	0.093	0.033	0.081	0.957	0.059	0.883
UHC service coverage index								
Coefficient	0.860 **	0.242	0.739 **	0.144	0.751 **	−0.105	0.773 **	−0.058
*p* value	0.000	0.198	0.000	0.448	0.000	0.581	0.000	0.759

* *p* < 0.05, ** *p* < 0.01. Overall refers to the absolute value of each country each year. Relative change represents the annual difference in each country each year.

## Data Availability

The data used in this study are from public and property databases.
